# Case report: Treatment-resistant schizophrenia with auto-aggressive compulsive behavior—Successful management with cariprazine

**DOI:** 10.3389/fpsyt.2023.1209077

**Published:** 2023-06-29

**Authors:** Lubova Renemane, Elmars Rancans

**Affiliations:** ^1^Riga Stradins University, Department of Psychiatry and Narcology, Riga, Latvia; ^2^Riga Centre of Psychiatry and Addiction Disorders, Riga, Latvia

**Keywords:** treatment-resistant schizophrenia, cariprazine, aggressive compulsive behavior, treatment of schizophrenia, antipsychotics

## Abstract

The present case report describes a patient with treatment-resistant schizophrenia and auto-aggressive compulsive behavior who was effectively treated with a third-generation antipsychotic medication, cariprazine. The diagnosis was made 12 years ago, and the patient has been hospitalized 14 times and undergone various antipsychotic treatments. Despite receiving both inpatient and outpatient care, the patient's response to treatment has been only partial, and he has been classified as a treatment-resistant case. Therefore, the patient was switched to cariprazine, which led to significant improvements in both positive and negative symptoms, as well as the complete reduction of auto-aggressive compulsive behavior. These improvements contributed to the patient's overall social functioning and the achievement of remission, while also avoiding polypharmacy and eliminating the metabolic side effects associated with previous treatments.

## 1. Introduction

Schizophrenia is a chronic psychiatric disorder affecting major thought processes such as perception, thinking and behavior. It is characterized by three major symptom domains: positive, negative and cognitive. Given these symptoms, schizophrenia has a major impact on the functioning and quality of life of patients, as they cause disruptions to work and school performance as well as to social relationships.

Studies estimate that about 30% of patients will develop treatment-resistant schizophrenia (TRS). The development of the concept of TRS was associated with the introduction of the second-generation antipsychotic drugs in dichotomic terms of response or no response to previous drug (1). The advancement of psychiatry science, using of integrated biopsychosocial approach and a multi-level assessment of treatment response lead to formulate concepts such as remission, much closer to the idea of recovery and functional recovery, based on patients' social functioning level (2). Up to now, TRS is defined as the presence of persisting symptoms despite two or more antipsychotic trials with adequate dose and treatment duration as well as documented medication-adherence (3). One of the defining features of TRS is the persistence of positive symptoms (4), yet cognitive or negative symptoms may also persist (5). Patients with TRS tend to have more severe symptoms, worse cognitive functioning and therefore higher costs to the healthcare providers (6). In addition, it further increases the burden on families and caregivers as they need to spend significant amount of their time and income on patient-care activates, having a negative impact on family life (6–8). It is important to note that TRS affects not only the patient and their family, but also the medical team responsible for their care. Healthcare professionals may develop negative attitudes such as pessimism, therapeutic nihilism, and lack of intellectual curiosity toward these patients (6). These patients come to be perceived and labeled as ‘difficult' (6, 9).

In order to provide clinical guidance on the identification and management of TRS, a research group was launched who developed and published a guideline (6). According to their view, re-evaluation of treatment plan is needed in case of non-response to two different antipsychotic medications including clozapine, consideration of adjunctive treatment with non-pharmacologic therapies and sharing of decision-making process among the treatment team, patient, and family, in order to choose the most adequate treatment plan. Finally, the authors suggest monitoring for potential markers of TRS such as high dose of antipsychotics, frequent hospitalisations, and ineffective polypharmacy.

Obsessive-compulsive symptoms in schizophrenia are clinically significant, may manifest in the prodromal or acute phase of the disorder, and have a prevalence rate of 12 to 20% (10, 11). Clinical studies report that obsessive-compulsive symptoms (OCS) not only increase the severity of symptoms and worsens the prognosis of schizophrenia but also causes the patient to respond poorly to conventional antipsychotic treatment, and generally have worse outcomes in social functioning (10–12).

Aggression is a significant concern in individuals with schizophrenia, and it can manifest in various forms, including auto-aggression as a compulsive symptom. According to recent meta-analysis data, the pooled prevalence of aggression is reported as 33.3% (13). Additionally, the estimated prevalence rates for verbal aggression, property-oriented aggression, auto-aggression, and physical aggression are 42.6, 23.8, 23.5, and 23.7%, respectively. In another cross-sectional study, several associated factors for aggressive behavior among patients with schizophrenia were identified, including male gender, unemployment, previous history of aggression, psychotic symptoms, drug nonadherence, poor social support, and alcohol use (14).

Scientists have discussed if this condition is a pure comorbidity or a subtype of schizophrenia. The term schizo-obsessive disorder refers to a clinical spectrum of disorders with characteristics of schizophrenia and obsessive compulsive disorder (OCD) (11, 12). Some studies have pointed out that schizo-obsessive disorder can be considered a subtype of schizophrenia and not a distinct clinical entity (11). Nevertheless, the OCD with psychotic features and absent insight is distinguished in the diagnostic and statistical manual of mental disorders fifth edition (DSM-5) (15). Similarly, the international classification of diseases 11th version (ICD-11) has specified insight levels for OCD as good to fair insight or poor to absent insight (16). Another aspect of relationships is iatrogenic OCS secondary to atypical antipsychotics, particularly clozapine, which appear to have a role in inducing or exacerbating OCS (11, 12, 17). Currently, there is no consensus on the management of OCS in TRS patients, but this does not mean that the condition cannot be improved, rather that these improvements may take time and experimentation.

Cariprazine is a third-generation antipsychotic with dopamine D_2_-D_3_ partial agonist with preferential binding to the D_3_ receptors (18). The exceptional receptor profile and tolerability is a potential beneficial value of this drug. Cariprazine is effective in the treatment of schizophrenia and may be particularly beneficial for the negative symptoms of this disorder. It is approved for the treatment for schizophrenia by the European Medicines Agency (19) and additionally for the treatment of depressive and manic/mixed episodes associated with bipolar I disorder by the Food and Drug Administration (20). It further showed efficacy in the adjunctive treatment of major depressive disorder (21). In schizophrenia, cariprazine showed statistically significant superiority in the treatment of acute patients (22–24) and in long-term relapse prevention (25) over placebo. Furthermore, it statistically significantly outperformed risperidone in the treatment of patients with persistent, predominant negative symptoms (26).

The present case report describes a TRS patient with auto-aggressive compulsive behavior that was managed successfully with switching to cariprazine.

## 2. Case presentation

### 2.1. Background history

A 35-year-old Caucasian male does not have a family history of mental illness or substance abuse disorder. Early developmental progression was without any delay. During adolescence, the individual experienced tension, decreased energy, and symptoms of agoraphobia, leading to three visits to a child psychologist. The individual later pursued vocational education and became a potter, and then worked part-time as an assistant of a social worker. He lived alone and had not been in a romantic relationship until his current episode of illness.

### 2.2. Interventions and their outcomes

The first notable complaince appeared when he was 23 years old. He developed tension, obsessive thoughts and auto-aggressive compulsive behavior, he used to beat walls with his fist. After a consultation with a psychiatrist he was diagnosed with obsessive-compulsive disorder according to DSM-5 (300.3) (15) and Sertraline 100 mg/day was prescribed. However, after 2 months of gradually increasing the dosage to 150 mg/day, there was no improvement in the patient's mental state. The patient's therapy was then switched to Clomipramine, starting with an initial dose of 150 mg and gradually increased to 300 mg/day. Despite 2 months of treatment, the patient's compulsive behavior was not controlled, and Paroxetine was prescribed with a gradual titration to 60 mg. After 2 months of treatment, Risperidone was added to the therapy at a dosage of 2 mg/day, but there was no significant improvement in the patient's clinical symptoms.

The patient became socially avoidant, experienced a decreased in motivation and ability to initiate and persist in self-directed purposeful activities, and started neglecting the hygiene. First psychotic symptoms manifested 2 months prior to being admitted to a psychiatric hospital, and characterized by auditory hallucinations, thoughts echo, thought insertion and delusion of control. Furthermore, he engaged in self-harming behavior by hitting himself in the face, which resulted in severe bruising.

Due to the above-described symptoms, the patient was admitted to the hospital. Subsequently, a structured psychiatric assessment interview was conducted, revealing that the patient met the criteria for Schizophrenia (295.90) as per the DSM-5. Diagnostic testing to exclude any organic, neurological, somatic or psychoactive substance etiology of psychosis were performed. Patient's vital signs, biochemical blood test, C-reactive protein, glucose level, total bilirubin, alanine aminotransferase, urea, urinalysis, and thyroid hormones were within normal limits. The rapid plasma reagin test, HIV serology, Hepatitis B and C surface antigen and urine drug tests were negative. Magnetic resonance imaging showed no pathology of the brain. No paroxysmal activity was registered on the electroencephalogram. A chest x-ray showed no abnormalities in the lungs. Psychometric psychological assessment revealed signs of endogenous type of mental disorders. Thinking process is characterized with lose of associations, paralogy, abstract reasoning was based on insignificant facts. No reductions were observed in sensory memory, short-term memory, and long-term memory. The personality was characterized by passive social withdrawal, emotional and social isolation. Neurological and somatic status were without abnormalities. Substance use disorder evaluations did not reveal any substance or alcohol dependence syndrome. The patient did not have any depressive, manic or hypomanic episodes; therefore, affective disorders were ruled-out.

At the inpatient psychiatry unit, the patient received haloperidol 15 mg/day, divalproate 1500 mg/day, trihexyphenidyl 6 mg/day and diazepam up to 20 mg/day in combination with rehabilitation. His symptoms partially improved and after discharge, the patient continued the outpatient treatment.

In total, the patient had 14 hospital admissions; the last one was in May 2021. The reasons for hospitalization were psychotic exacerbations, safety reasons due to aggressive compulsive behavior, and decline in social functioning. He was involved in out-patient care, and demonstrated good compliance, and adherence to recommended treatment regimens. Throughout this period of the illness, auditory hallucinations, thoughts echo, thought insertion, delusions, social withdrawal, motor retardation, poor attention, disturbance of volition, poor impulse control, auto-aggressive behavior and decline in social functioning were persisted. Over the past 12 years, different treatment schemes were applied. For at least 4 months, he received the following therapy regimes in these years: olanzapine 40 mg per day; risperidone 6 mg, trihexyphenidyl 6 mg; risperidone up to 6 mg, trihexyphenidyl up to 8 mg, clozapine 200 mg per day; olanzapine 20 mg, haloperidol 10 mg, buspirone 30 mg, trihexyphenidyl 6 mg per day; haloperidol 15 mg, clozapine 150 mg, trihexyphenidyl 6 mg per day; clozapine 400 mg, mirtazapine 15 mg, divalproate 1,000 mg daily; amisulpiroid 600 mg, clozapine 75 mg, divalproate 1,500 mg, trihexyphenidyl 6 mg per day. Clozapine up-titration was impossible due to side effects such as sedation, hypersalivation and weight gain. The patient refuses to receive long-acting injectable antipsychotics. Despite ongoing treatment, the patient achieving only a partial response in terms of above-described symptoms. An overview of inpatient and outpatient pharmacological treatments 12-year time line summery listed in Table 1.

**Table 1:** Inpatient and outpatient pharmacological treatments attempt 12-year timeline summary with duration of treatment at least 4 months.

**Duration of treatment**	**Pharmacological treatment/Daly dose**	**Comments**
7 months	Haloperidol 15 mg Divalproate 1,500 mg	Trihexyphenidyl 6 mg/day was administrated to manage parkinsonism. Diazepam up to 20 mg was administrated for 2 months to manage anxiety. Partial response to treatment.
4 months	Olanzapine 40 mg	Partial response to treatment.
4 months	Risperidone 6 mg	Trihexyphenidyl 6 mg/day was administrated to manage parkinsonism. Partial response to treatment.
7 months	Risperidone 6 mg Clozapine 200 mg	Trihexyphenidyl 8 mg/day was administrated to manage parkinsonism; Clozapine up-titration was impossible due to side effects such as sedation, hypersalivation and weight gain. Partial response to treatment.
8 months	Olanzapine 20 mg Haloperidol 10 mg Buspirone 30 mg	Trihexyphenidyl 6 mg/day was administrated to manage parkinsonism. Partial response to treatment.
7 months	Haloperidol 15 mg Clozapine 150 mg	Trihexyphenidyl 6 mg/day was administrated to manage parkinsonism; Clozapine up-titration was impossible due to side effects such as sedation, hypersalivation and weight gain. Partial response to treatment.
6 months	Clozapine 400 mg Mirtazapine 15 mg Divalproate 1,000 mg	Clozapine up-titration was impossible due to side effects such as sedation, hypersalivation and weight gain. Partial response to treatment.
9 months	Amisulpiroid 600 mg Clozapine 75 mg Divalproate 1,500 m,	Trihexyphenidyl 6 mg/day was administrated to manage parkinsonism; Clozapine up-titration was impossible due to side effects such as sedation, hypersalivation and weight gain. Partial response to treatment.
8 months	Amisulpride 400 mg Clozapine 100 mg Mirtazapine 30 mg	Trihexyphenidyl 4 mg/day was administrated to manage parkinsonism; Clozapine up-titration was impossible due to side effects such as sedation, hypersalivation and weight gain. Partial response to treatment. The patient was switched to cariprazine from this therapy.

The patient was treated on an outpatient basis in January 2022 with a medication regimen that included amisulpride 400 mg/day, clozapine 100 mg/day, mirtazapine 30 mg/day, and trihexyphenidyl 4 mg/day. Despite treatment, the patient continued experiencing severe auditory hallucinations, thought insertion, social withdrawal, motor retardation, disturbance of volition, poor impulse control, and auto-aggressive behavior. He engaged in self-mutilation by hitting his own face, which caused subcutaneous hemorrhage. Assessment of total score on the Positive and Negative Syndrome Scale (PANSS) (27) was 86 with positive sub-score of 15, negative sub-score of 24 and general sub-score of 47. The Global Assessment of Functioning (GAF) (28) score was 20, and the Yale-Brown Obsessive Compulsive Scale (Y-BOCS) (29) showed a score of 22 that indicates severe level of symptoms. His body mass index (BMI) was 30.5 kg/m^2^ (height = 190 cm, weight = 110 kg).

Due to persistent symptoms and low functioning, the patient's medication regimen was changed without cross-titration to cariprazine 6 mg/day, clozapine 100 mg/day and mirtazapine 30 mg/day. In the first 2 weeks, the patient experienced extrapyramidal side effects in the form of a moderate tremor in his fingers, thus trihexyphenidyl 4 mg/day was prescribed. Otherwise, patient showed good tolerability. A time line summary of pharmacological treatment, clinical picture and scores of psychometric scales after switching to cariprazine presented in Table 2.

**Table 2:** Outpatient care timeline summery of pharmacological treatment, clinical picture and psychometric scales.

**Date Duration**	**Pharmacological treatment**	**Description of clinical presentations**	**PANSS, GAF, Y-BOCS, BMI**
Jan 2022 Baseline	Cariprazine 6 mg Clozapine 100 mg Mirtrazapine 30 mg EPS after 2 weeks Trihexyphenidyl 4 mg	Severe auditory hallucinations, thought insertion, social withdrawal, motor retardation, disturbance of volition, poor impulse control, auto-aggressive behavior. Severe impairment in personal grooming. He stopped brushing his teeth, playing the guitar, leaving the house, going to work or doing sports. Tension accompanied by an irresistible desire to hit himself. He engaged in self-mutilation and bet himself in the face, leading to subcutaneous hemorrhage. In order to avoid self-harm, the patient lied on his bed all day.	PANSS = 86 GAF = 20 Y-BOCS = 22 BMI = 30.5 kg/m2 High = 90 cm Weight = 110 kg
Mar 2022 8 weeks	Cariprazine 6 mg Mirtrazapine 15 mg Trihexyphenidyl 4 mg	Moderate auditory hallucinations, thought insertion, social withdrawal, motor retardation, disturbance of volition, poor impulse control, auto-aggressive behavior (partial control). The patient was finally able to get out of his bed, go outside and started working two times a week.	
May 2022 16 weeks	Cariprazine 6 mg Mirtrazapine 15 mg Trihexyphenidyl 4 mg	Mild auditory hallucinations, social withdrawal, motor retardation, disturbance of volition, poor impulse control, auto-aggressive behavior (partial control). The patient was able to play the guitar for 20 mins and started boxing with a trainer for three times a week.	
Jun 2022 20 weeks	Cariprazine 6 mg Trihexyphenidyl 4 mg	Absent of auditory hallucinations, thought insertion and autoagressive behavior. Started working full-time at a woodworking manufacturer. He was glad to be able to do sports and play the guitar again, although for a limited amount of time.	
Jul 2022 24 weeks	Cariprazine 6 mg Trihexyphenidyl 4 mg	The patient began to become socially active again, rarely experiences tensions and the urge to hit himself, he does not engage in aggressive behavior and his auditory hallucinations seized. The patient was able to do sports and play the guitar without any time-limitation.	PANSS = 43 GAF = 67 Y-BOCS = 3 BMI = 26.3 kg/m2 High = 190 cm Weight = 95 kg

PANSS, Positive and Negative Syndrome Scale; GAF, Global Assessment of Functioning; BMI, body mass index; Y-BOCS, Yale-Brown Obsessive Compulsive Scale; EPS, extrapyramidal symptoms.

After 2 months, the severity of auditory hallucinations, thought insertion, social withdrawal, motor retardation, disturbance of volition and poor impulse control decrease to moderate level. He developed partial control of auto-aggressive behavior. The patient started working two times a week. Due to the improvement, clozapine was discontinued, and the dose of mirtazapine was decreased to 15 mg/day.

Four months after the first administration of cariprazine 6 mg/day, the severity of positive and negative symptoms were defined as mild. The patient reported interest in hobby, communication with acquaintances, and started physical activities.

Another month later, auditory hallucinations and thought insertion disappeared. He had full control of auto-aggressive behavior. This improvement enabled mirtazapine to be discontinued too.

**Figure 1:**
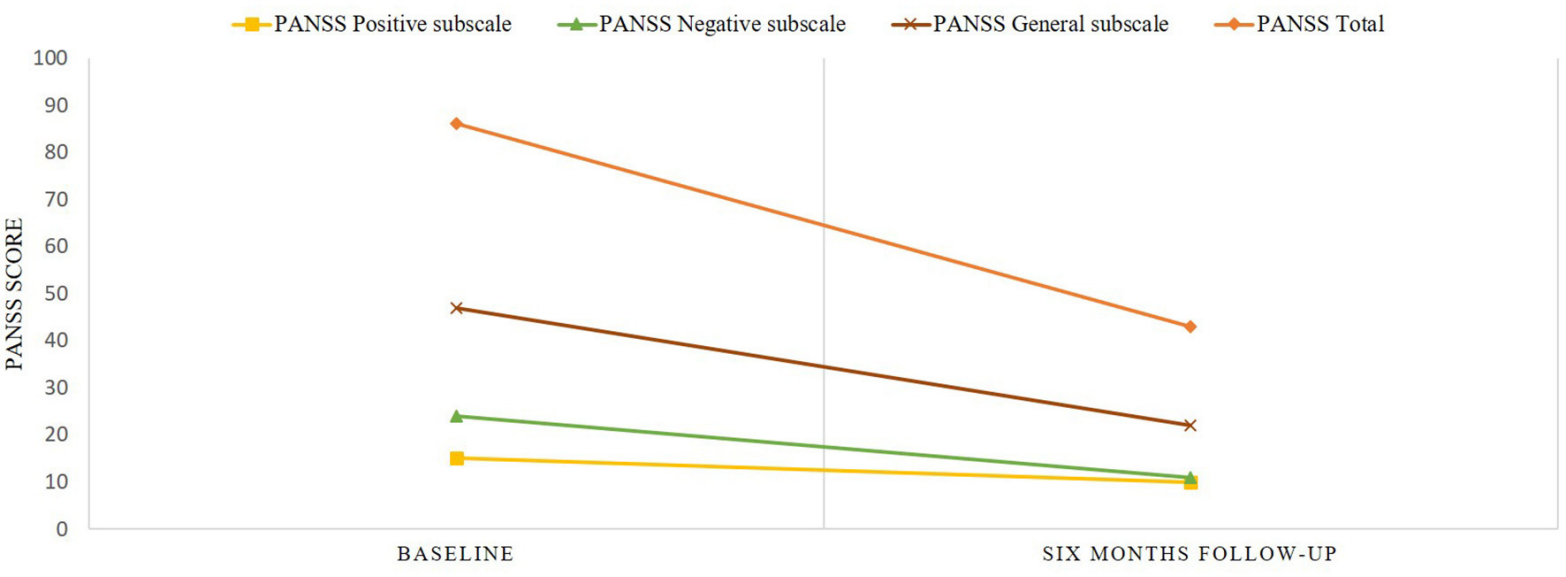
Changes of PANSS scores prior and after initiation of the treatment with cariprazine.

After 6 months of treatment with cariprazine 6 mg/day, the patient began to become socially active again, he demonstrated absent of auditory hallucinations and thought insertion, and he does not engage in auto-aggressive behavior. At this point, the PANSS total score was 43 with positive sub-score of 10, negative sub-score of 11 and general sub-score of 22, meaning an overall reduction of 50%. The GAF score was 67, which is fairly good as a score of ≥60 points is considered as demonstrating adequate functioning (30). The Y-BOCS score decreased to 3, and the patient's BMI to 26.3 kg/m^2^. The PANSS scores prior to switching to cariprazine and after 6 months of therapy with cariprazine presented in Figure 1.

## 3. Discussion

The disorder manifested in adolescence and retrospectively, a prodromal phase of schizophrenia can be detected with neurosis-like symptoms such as tension, anxiety, decreased energy and agoraphobia. The acute phase start at the age of 23, characterized by auditory hallucinations, thoughts echo, delusion of passivity, obsessive thoughts, tension and aggressive compulsive behavior. Throughout the course of the disorder, the patient developed passive social withdrawal, motor retardation, poor attention, disturbance of volition, poor impulse control and active social avoidance. The patient had 14 readmission to mental hospitals, often because he were discharged before he had adequately recovered, that describe the revolving door phenomenon (31). This highlights the importance of providing comprehensive and effective treatment and support to individuals with mental illness to prevent the revolving door phenomenon from occurring.

Despite receiving continuous pharmacological treatment in the form of both first- and second-generation antipsychotics in various combinations, it exemplifies a “difficult to treat” condition. The treatment response was only partial, and complete remission was not achieved. The case meets the criteria for treatment-resistant schizophrenia (TRS) as per the consensus guidelines of the Treatment Response and Resistance in Psychosis Working Group, given the reasons outlined above (5). The patient had active and persistent symptoms for over 12 weeks with impaired functioning and at least 6 weeks of treatment with an appropriate usage of two antipsychotics from different drug profiles at doses equivalent to over 600 mg chlorpromazine per day, and he was also compliant.

Throughout the course of schizophrenia, different approaches of pharmacological treatment were tried without sufficient improvement. Clozapine is wellknown and defined as the first-line medication for the management of TRS with evidence from several studies (32). For instance, the patient did not response to clozapine 400 mg/day and the up-titration was impossible due to side effects such as sedation, hypersalivation and weight gain. In terms of cariprazine, there is some evidence supporting its effectiveness in TRS patients, however it is only based on case reports (33–35). While both cariprazine and clozapine have affinity for dopamine D2 and D3 receptors, their receptor profiles differ in terms of partial agonism vs. antagonism. Clozapine primarily acts as an antagonist at multiple receptors, including dopamine D1, D2, D3, and D4 receptors, serotonin 5-HT2A, 5-HT2C, 5-HT3 receptors, and histamine H1 receptors (32). Cariprazine, on the other hand, acts as a partial agonist at dopamine D2 and D3 receptors and as an antagonist at serotonin 5-HT2A receptors (18). While clozapine exhibits broader antagonistic effects on serotonin receptors, both medications antagonize 5-HT2A receptors. Similarities in receptor profiles can potentially be associated with comparable treatment efficacy. Another possible explanation for the effectiveness of cariprazine in this case could be attributed to a comparison of dose equivalents between cariprazine 6 mg and clozapine 400 mg, where the dose equivalent for cariprazine is ~1.5 times higher (36).

Unfortunately, the use of clozapine in schizophrenia patients with obsessive-compulsive behavior could lead to the worsening of OCS (11, 12, 17). Indeed, in the present case, such symptoms of the patient did not change significantly during clozapine treatment according to the medical records as monitored by standardized rating scales.

Several studies have provided evidence of aripiprazole efficacy in improving symptoms and achieving remission in patients with TRS (37, 38). Additionally, several case reports demonstrated effective use of aripiprasole in OCD (37). Aripiprazole and cariprazine share similarities in their receptor profiles, particularly in their partial agonist activity at dopamine D2 and D3 receptors. This partial agonism may contribute to their ability to stabilize dopamine activity in the brain. Additionally, both aripiprazole and cariprazine display antagonistic effects on serotonin 5-HT2A receptors, which may further contribute to their overall pharmacological effects.

Although a recent study summarizing evidence from clinical trials and real-world studies recommend cross-titration in case of switching from another antipsychotic to cariprazine (39), in this case the authors decided to switch from amisulpride 400 mg/day to cariprazine 6 mg/day without cross-titration due to the severity of symptoms. This abrupt switching strategy was well tolerated by the patient.

Based on the results, the present case demonstrates the efficacy of cariprazine in the treatment of psychotic, negative symptoms and self-aggressive compulsive behavior in a TRS patient. After 6 months of therapy, 50% of the PANSS reduction. The GAF improvement was detected and indicates adequate day-to-day functioning, which was unimaginable previously. Reviewing the literature, this is also supported by other studies focusing on the efficacy of cariprazine in hostility (40, 41). For instance, a recent *post-hoc* analysis looked at the pooled data from three randomized, placebo-controlled, phase 2/3 studies with patients who had acute exacerbation of schizophrenia and found that with cariprazine there was a significant improvement in hostility compared to patients treated with placebo as measured by the PANSS-derived Marder hostility subscale (40).

Another important aspect in this case is the prevention of further polypharmacy—the combination of more than one antipsychotics and/or antidepressants—with cariprazine. After the fifth month of cariprazine treatment, the patient stopped any other medication and received only cariprazine as monotherapy.

Since the patient received the highest recommended dose, 6 mg/day, a side-effect in the form of hand tremor was induced which was then compensated with anticholinergic medication. Otherwise, the medication was welltolerated. Another safety aspect in this case was metabolic symptoms. It is a wellknown fact that TRS patients belong to a high-risk group of developing metabolic syndrome due to the widespread use of polypharmacy treatment with second-generation antipsychotics as well as due to general lifestyle (40). Given the fact that cariprazine is metabolically neutral, the patient lost 15 kg body weight and his BMI became close from class obese to only class overweight. Moreover, clozapine and mirtazapine, medications that have high metabolic risk, were discontinued and with the reduction of negative symptoms, the patient changed his lifestyle and became more active. It is important to note that the patient did not change his eating regime or the amount of food intake.

Finally, given the fact that the switch to cariprazine prevented further hospitalization of the patient, this case reduced the healthcare costs of the provider as well.

All in all, this is an example of continuous treatment efforts and maintenance of therapeutic optimism that finally resulted in symptom improvement. Nonetheless, it is crucial to continue to follow up the patient in the future in order to understand further changes in symptoms, control tolerability and social functioning. More evidence is needed regarding the long-term effectiveness of cariprazine in TRS schizophrenia with auto-aggressive compulsive behavior.

## Data availability statement

The raw data supporting the conclusions of this article will be made available by the authors, without undue reservation.

## Ethics statement

Ethical review and approval was not required for the study on human participants in accordance with the local legislation and institutional requirements. Written informed consent was obtained from the patient to publish this case.

## Author contributions

LR and ER contributed to the design and conception of the manuscript and data analysis and revision of subsequent versions. LR wrote the first draft of the manuscript. Both authors contributed to the article and approved the submitted version.
